# Served Well? A Pilot Field Study on the Effects of Conveying Self-Control Strategies on Volleyball Service Performance

**DOI:** 10.3390/bs9090093

**Published:** 2019-09-02

**Authors:** Maik Bieleke, Claudio Kriech, Wanja Wolff

**Affiliations:** 1 Department of Psychology, University of Konstanz, 78464 Konstanz, Germany; 2 Department of Empirical Educational Research, University of Konstanz, 78464 Konstanz, Germany; 3 Department of Sport Psychology, University of Konstanz, 78464 Konstanz, Germany; 4 Department of Educational Psychology, Institute of Educational Science, University of Bern, 3012 Bern, Switzerland

**Keywords:** self-control, goal setting, if-then planning, implementation intention, volleyball serve, coach instructions

## Abstract

Volleyball serves constitute an important example for a self-controlled sequence of actions in sports that is difficult to improve. It is therefore paramount to investigate whether and how conveying self-control strategies to athletes affects their service performance. To address this question, we conducted a pilot field study with sixty-two players from four Swiss volleyball schools. They performed a warm-up and subsequently a first series of 15 serves. Objective service performance was measured in terms of errors, velocity, and precision. Afterwards, players formulated either individual goals (goal condition) or plans (plan condition) based on their coaches’ correction instructions. In a second series of 15 serves objective performance was worse in some respects compared to the first series (i.e., more errors in the plan condition, reduced precision in both conditions). Mixed-effects analyses of performance development across conditions in the second series showed initially reduced but steadily recouping precision and velocity, while the number of errors stayed constant. In contrast to the objective performance, coaches evaluated their players’ service performance during the second series of serves as substantially better than during the first series. Taken together, the results of this pilot field study suggest that conveying either goals or plans as self-control strategies may involve initial adjustment costs followed by a subsequent recovery period.

## 1. Introduction

Many sports involve closed-skill actions that can be performed by athletes without the influence of an opponent (i.e., in a self-controlled and self-paced manner) rather than being performed in response to an external event (i.e., in an environmentally controlled and externally paced manner) [1,2]. Serves that bring the ball into play (e.g., in volleyball and tennis) are prototypical examples of such self-controlled actions. Successful serves can lead to immediate point gains, while service errors lead to loss of points. In addition, the quality of the serve (i.e., velocity and precision) can be crucial for succeeding in the subsequent rally, as it determines how difficult it is for the opponent to receive the serve and to structure the own game. Accordingly, the tactical focus on scoring points through one’s own serves in volleyball is increasing [3].

However, an optimal serve requires the smooth initiation of a complex sequence of various motor activities. At least five separate movement phases can be differentiated in volleyball serves (i.e., windup, cocking, acceleration, deceleration, and follow-through), which together take less than two seconds [4]. The corresponding biomechanical processes must first be acquired and then continually improved [5]. For instance, volleyball players gradually develop better coordination patterns of their shoulder, elbow, and wrist movements as they gain service expertise, resulting in improved patterns that differ substantially from the coordination patterns observed among novice players [6]. Improving these coordination patterns is not only relevant from a success maximization perspective: electromyographic analyses show that substantial forces act on the shoulder area during service [7], and poor coordination can thus lead to serious injuries in this area particularly [8]. Thus, both success and injury prevention call for improving the serving performance as a central training objective.

Volleyball serves thus constitute an important example for a performance that is essentially self-controlled and yet difficult to improve. This raises the question of whether and how conveying specific self-control strategies to athletes might affect their service performance [9]. Self-control strategies are defined as targeted adjustments of one’s actions in order to obtain a goal [10]. This involves either the prevention of an unwanted response (e.g., jumping off too early or too late after tossing up the ball) or the elicitation of a wanted response (e.g., adopting a greater stride length if the ball is too far away). From this perspective, any action involved in a volleyball serve can be adjusted by means of self-control strategies. It is not obvious, however, whether conveying self-control strategies to athletes affects their service performance for better or for worse, or whether there is no effect on performance at all (e.g., because the biomechanical sequence has been automatized by training). Research on self-controlled action is commonly limited to artificial laboratory tasks, leaving gaps in our knowledge about how self-control strategies might affect complex behaviors in more naturalistic settings. Addressing this issue with a focus on volleyball serves is paramount for training and competition as coaches should be aware of both positive and negative effects on performance when conveying self-control strategies to their players.

One fundamental strategy is setting goals or goal intentions [11], defined as specifying desired end states that a person attempts to reach. Goals emphasize the execution of actions or the achievement of outcomes and typically have the format "I want to perform action A / obtain Outcome O!". Setting goals commonly increases the probability of successfully implementing actions [12]. In the domain of sport, too, the effectiveness of goals has been demonstrated [13,14]. A second, more elaborate self-control strategy is the formulation of if-then plans (implementation intention) [15,16] in addition to a goal, which specifies exactly how one wants to reach the desired end state. Specifically, a plan connects an action with the occurrence of a specific situation: "If situation S occurs, then I will perform action A!". Making plans raises the probability of successfully performing an action [16,17]. However, while planning is a promising strategy in sports, its effectiveness for improving performance remains to be explored [18]. 

Goal setting and planning are particularly suited for investigating the effects of providing self-control strategies in applied sport settings. First, both goal setting and planning are well-established strategies in the self-control literature and consistently benefit performance across various domains [17]. Second, goals and plans are simple strategies that should be easily conveyed to athletes without requiring an extensive intervention [18]. Third and related, it seems likely that many coaches provide goals and plans as self-control strategies without necessarily being aware of it. For instance, instructions to "toss the ball higher" or "if you have tossed the ball too far in front of you, then increase your stride length" are essentially goals and plans, respectively, according to the above definitions. Thus, it is paramount to investigate whether and how conveying these strategies to athletes affects their performance. 

Fortunately, the extensive research on goals and plans allows to derive specific and testable predictions in the context of volleyball serves. While both goals and plans typically benefit performance, planning is commonly even more effective than goal setting across domains [16,17]. It is therefore plausible to expect generally better service performance when if-then plans are conveyed to athletes compared to goals. Moreover, goals and plans have consistently been shown to differ in the extent to which they allow an action to be performed automatically e.g., neuroimaging studies [19,20,21], which permits hypotheses regarding the temporal development of performance after goal setting and planning. Specifying an action as a goal is assumed to instigate top-down processes that require continuous attention and cognitive processing because the when, where, and how of its execution are not specified [19,22]. According to this argument, athletes who are instructed to set goals to perform a certain action during service should need to explicitly attend to and think about performing these actions. This might withdraw their attention from other important actions during the serve, akin to the effects of conscious intrusions during the performance of well-trained actions (i.e., conscious reinvestment) [23]. As a consequence, setting a goal might initially hamper performance. However, it might improve in the long run as the intended action is repeatedly executed and therefore becomes more and more automatic (i.e., a practice effect), requiring less expenditure of conscious attention and cognitive resources.

In contrast, if-then planning is assumed to facilitate an earlier (or even immediate) automation of actions. This assumption rests on research showing that the "if (situation) - then (action)" format of plans makes the planned situation easier to recognize when it occurs [24,25] and automates the initiation of the planned action [26,27]. This automaticity has been explained in terms of a joint sensorimotor simulation of situation-action links involved in planning that facilitates later execution of actions when the situation is encountered [28], akin to the sensorimotor simulation of actions in sports [29]. Given that planning automates the execution of an action, more attentional and cognitive resources should remain for executing other actions required during the serves. Compared to goals, it can thus be expected that planning does initially have less detrimental effects on performance and thus leads to more rapid performance improvements. 

To sum up, we examine whether and how conveying the self-control strategies of setting goals and making plans to athletes affects their service performance. This sheds novel light on the potential effects of self-control strategies on complex behaviors in naturalistic settings and might help athletes and trainers gauge the impact of these self-control strategies on performance. Specifically, we investigate aggregate effects on errors, velocity, and precision as objective indicators of performance, as well as the temporal development of these performance indicators immediately after setting a goal or making a plan. To this end, we conducted a pilot field study in which active beach volleyball players from four Swiss volleyball schools were recruited. Athletes performed two series of 15 serves with either a goal or a plan to improve certain actions after the first series. Besides objective performance indicators, we also assessed the coaches’ ratings of performance improvement.

## 2. Materials and Methods

### 2.1. Participants

A total of 27 female and 35 male volleyball players from four volleyball schools in Switzerland were recruited for the study (age: *M* = 13.9 years, *SD* = 1.0, *Min* = 11, *Max* = 16). Participation was voluntary and we obtained informed consent from the players and their parents prior to data collection. The players were randomly assigned to a goal (*n* = 29) or an implementation intention condition (*n* = 33). This sample size is sensitive to small-to-medium effects in analyses across players in the two conditions (*d* = 0.32) and to medium-to-large effects in comparisons between the two conditions (*d* = 0.72) at a test power of 80% and an α error probability of 5%. Conditions did not differ regarding age, *t* (60) = 1.54, *p* = 0.128, *d* = 0.39, 95% CI [−0.12, 0.90], or gender, χ² (1), *p* = 0.849.

On average, the athletes had been playing beach volleyball for *M* = 3.7 years (*SD* = 1.80, Min = 1, Max = 8). They reported to practice *M* = 11.7 hours per week (*SD* = 5.5, *Min* = 5, *Max* = 40), a reasonable amount of training for adolescent athletes [30]. Thirty-six players (58.1%) stated that they normally used the jump float serve, the others reported to use the float serve. Eighteen players engaged in a second sport (29.0%) and spent *M* = 2.4 hours per week on it (*SD* = 1.9, *Min* = 1, *Max* = 8). Sixteen players (25.8%) reported experience with psychological training. *t*-tests for continuous and chi-square tests for categorical variables showed no difference between the goal and the plan condition for these or demographic data, *p*s > 0.10.

### 2.2. Materials and Instruments

#### 2.2.1. Objective Performance

Objective service performance was measured with three different indicators: errors, velocity, and precision. We determined errors according to official beach volleyball rules (e.g., if the ball was driven on the ground outside the boundary lines or in the net) [31]. Velocity was determined using a Bushnell Speedster III sports radar gun [32] and operationalized as the maximum velocity reached by the ball while crossing the field (in km/h). The device can measure velocities between 15 and 180 km/h with a precision of ± 2 km/h. The feasible distance for an object of the size of a baseball is 27 m; measuring the velocity of a volleyball over the 16 m length of a beach volleyball field is therefore not a problem. Precision was assessed with a measuring tape and operationalized as the distance between the position at which the ball hit the ground and the target position. The target position was always defined as the center of opponent team’s end line because this position represents an important conflict zone for the opponent team’s reception [33] and thus provides a high probability of scoring an ace or at least makes it more difficult for the opponent to receive the ball and to set up their own game.

#### 2.2.2. Coach Ratings

Besides the objective performance indicators, we also assessed the coach’s ratings of performance improvements. The coach observed both series of serves (i.e., before and after the intervention) and was afterwards asked to evaluate the change in serving performance on a scale from −5 (much worse) over 0 (same) to +5 (much better).

#### 2.2.3. Final Questionnaire

At end of the study, participants provided information about their demographics (age and gender) and their sports and training habits (e.g., volleyball training per week). In addition, their motivation to perform well was assessed with one item ("I was motivated to attain the goal in the best possible way") on a 5-point Likert scale (1 = not at all, 2 = almost not, 3 = neither nor, 4 = a little, 5 = very much).

### 2.3. Procedure

The study was carried out in accordance with the Helsinki Declaration of 1975 and with the approval of the local ethics committee (approval code: IRB 19-036). It began after a general warm-up under the supervision of the coach, which is a crucial element of any sport that requires fine motor skills [34]. Each player was then tested individually and started with a first series of 15 serves. The setup involved one experimenter, one measurement assistant, the coach and the player. Both the experimenter and the coach were located behind the service zone from which the serve was performed. The measurement assistant was positioned behind the opposite end line at the other side of the field. Players could perform their serves from anywhere in the service zone and with any type of serve, but they had to stick to the chosen service position and type throughout the study. The target position was always the middle of the opponent team’s end line. For each serve, objective performance indicators (i.e., errors, velocity, and precision) were assessed and recorded by the measurement assistant.

After warm-up and a baseline series of 15 serves, the experimenter asked the coach to identify one specific action that showed greatest potential for performance improvement and to provide a corresponding correction instruction. To make sure that this information could be conveyed to the players as a self-control strategy (rather than as external control or coach feedback), this exchange between coach and experimenter took place in the absence of the player. For the same reason, the experimenter then carried out the intervention alone with the player. Conveying goals and plans like this is a common experimental manipulation in research on self-control strategies [17].

Players were randomly assigned to the goal or the plan condition according to a randomized list of conditions. The experimenter instructed players in the goal and plan condition to form a goal intention (i.e., "My goal is to do Y") or an implementation intention (i.e., "If X occurs, then I will do Y"), respectively, based on the correction instruction. Accordingly, goals and implementations varied from player to player depending on this instruction. They focused, for instance, on tightening the hand or wrist (e.g., goal: "My goal is to have my wrist tight on the next serves."; plan: "When I serve, then I tighten my hand and fingers."), on adjusting steps and the stride length (e.g., goal: "I want to stay more stable by just taking one step before service."; plan: "When I approach the ball, then I take a small step first."), or on how to throw the ball (goal: "My goal is to throw the ball further forward on the next serve."; plan: "When I make the service, then I throw the ball higher up."). Care was taken to ensure that players formulated goal and implementation intentions in an effective way, for instance avoiding negations which have been shown to impair performance [35]. To check that players specified goals and plans as intended, they were asked to write them down on a sheet of paper. Importantly, coaches were not informed about the assignment to the goal versus plan condition. 

Immediately after the intervention was completed, the second series of 15 serves started. At the end of the second series, we asked the coach to rate performance improvements from the first to the second series of serves. After all players had been tested, they completed a final questionnaire and were debriefed together with the coach.

### 2.4. Data Analysis

Data analysis was performed using the statistical software R version 3.5.0 (R Core Team, Vienna, Austria) [36]. One focus was on how goal and implementation intentions affected aggregate performance (i.e., average errors, velocity, and precision in each series). This was analyzed with 2-within (series of serves: first vs. second) × 2-between (condition: goal vs. plan) mixed-factorial ANOVAs. Significant interaction effects were followed up with *t*-tests. To evaluate effect sizes, we determined partial eta-squared ($\eta_{p}^{2}$) for ANOVAs and Cohens *d* for *t*-tests along with the 90% and 95% confidence intervals, respectively. 

A second focus was on the development of performance especially during the second series of serves. We subjected the three performance indicators to generalized (errors) and linear (velocity, precision) mixed-effect models. We specified condition (goal vs. plan), serve number (1 to 15), and the condition × serve number interaction as fixed-effects. Random intercepts and slopes were estimated for each player to account for individual differences in initial performance level (intercept) and development (slope). Significance was evaluated using Satterthwaite-approximated degrees of freedom. 

The analysis of performance motivation and coach ratings of performance was conducted with independent and one sample *t*-tests. All tests were two-sided and the significance level was set to 0.05.

## 3. Results

### 3.1. Performance Motivation

Participants in the goal (*M* = 4.4, *SD* = 0.8) and plan condition (*M* = 4.6, *SD* = 0.6) were similarly motivated to perform well, *t*(60) = 1.00, *p* = 0.321, *d* = 0.25, 95% CI [−0.25, 0.76].

### 3.2. Aggregate Performance

**Figure 1:**
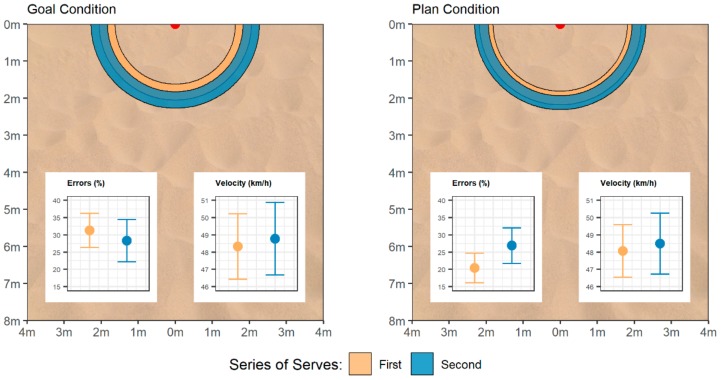
Performance in the first and second series of serves, separately for (**a**) the goal and (**b**) the plan condition. The figure shows a schematic depiction of the 8m × 8m court to which players served the ball. The target point at the center of the end line is marked red. The semicircles at the top depict precision (i.e., the average distance from the target point) as 95% confidence intervals around average precision. The diagrams show average errors and velocities along with their 95% confidence intervals.

We first aggregated the three performance measures by computing average errors, velocity, and precision across the 15 serves of both series of serves. For errors, a significant main effect of condition emerged, *F*(1, 60) = 5.10, *p* = 0.028, $\eta_{p}^{2}$ = 0.078, 90% CI [0.005, 0.199], qualified by an interaction with the series of serves, *F*(1, 60) = 4.25, *p* = 0.044, $\eta_{p}^{2}$ = 0.066, 90% CI [0.001, 0.183]. This interaction reflects significantly fewer errors in the first series of serves in the plan (*M* = 3.1, *SD* = 1.8) compared to the goal condition (*M* = 4.7, *SD* = 2.0), *t*(60) = 3.42, *p* = 0.001, *d* = 0.87, 95% CI [0.32, 1.41], whereas no difference between conditions emerged in the second series of serves (plan: *M* = 4.0, *SD* = 2.2; goal: *M* = 4.2, *SD* = 2.4), *t*(60) = 0.36, *p* = 0.719, *d* = 0.09, 95% CI [−0.41, 0.59]. That is, we found condition differences before the intervention and these differences vanished after the intervention. Regarding precision, a main effect of the series of serves emerged, *F*(1, 60) = 8.90, *p* = 0.004, $\eta_{p}^{2}$ = 0.129, 90% CI [0.025, 0.261], reflecting higher precision in the first series of serves (*M* = −194 cm, *SD* = 114) compared to the second (*M* = −210 cm, *SD* = 120). Other effects were not significant, *p* > 0.22. Finally, we found no significant main effects nor an interaction effect in the analysis of velocity, *p*s > 0.75. These results are depicted in Figure 1 and can be summarized as follows: performance was hampered in the second compared to the first series of serves with respect to service errors (plan condition; left bar graph at the bottom of the right panel) and with respect to precision (goal and plan condition; semicircles at the top of each panel), while velocity was similar across series and conditions (right bar at the bottom of each panel).

### 3.3. Development of Performance

#### 3.3.1. First series of Serves

In the first series of serves, a main effect of condition emerged for service errors, χ²(1) = 9.00, *p* = 0.003. Consistent with the analysis reported above, fewer errors were observed in the plan (*M* = 20.4%, *SD* = 12.0) compared to the goal condition (*M* = 31.3%, *SD* = 13.0). Other effects were not significant, *p* > 0.15. Accordingly, performance was stable during the first series of serves, as neither increases nor decreases in performance occurred over trials.

#### 3.3.2. Second Series of Serves

**Figure 2:**
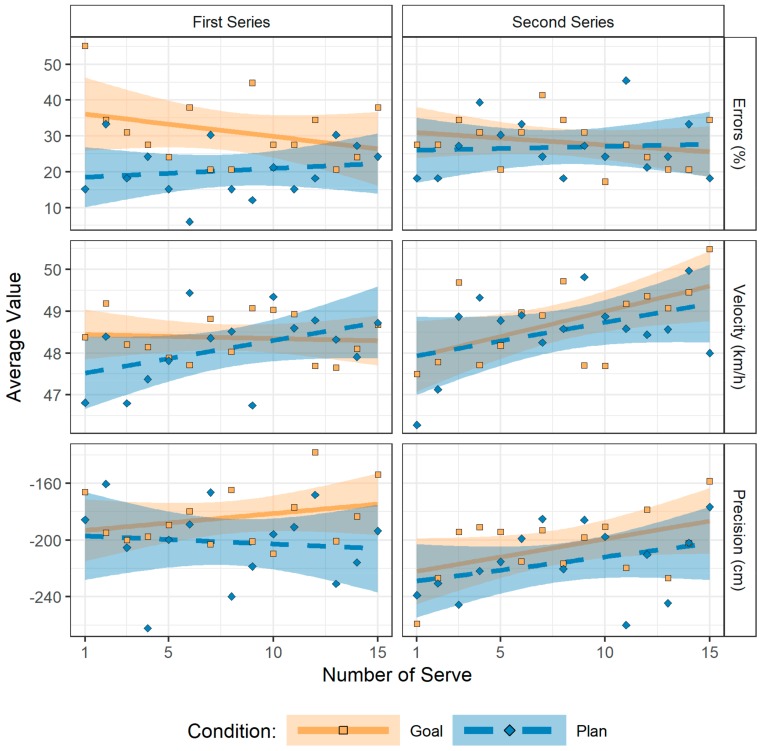
The development of performance during the first and the second series of serves. Performance was stable during the first series of serves. During the second series, there were drops in performance regarding velocity and precision with subsequent recovery. Dots represent average values per condition and number of serve, lines represent an ordinary least square regression line through these points along with a 95% confidence interval.

In the second series of serves, we found significant main effects of serve number (1 to 15) for velocity, *F*(1, 60.10) = 5.34, *p* = 0.024, and precision, *F*(1, 358.92) = 5.23, *p* = 0.023. Other effects were not significant, *p* > 0.36. These results reflect that velocity increased by about 5% over the 15 serves (first serve: *M* = 46.9 km/h, *SD* = 7.1; last serve: *M* = 49.2 km/h, *SD* = 6.5), which was accompanied by a 1/3 increase in precision (first serve: *M* = −247.8 cm, *SD* = 124.9; last serve: *M* = −169.2 cm, *SD* = 88.1). Figure 2 suggests that the aggregate decrease in precision reported above reflects an initial drop in performance immediately after the intervention which is followed by subsequent recovery. This is in line with a comparison of precision in the last serve of the first series and the initial serve of the second series among participants, *t*(36) = 3.82, *p* = 0.001, *d* = 0.63, 95% CI [0.27, 0.98]. Regarding velocity, the drop from the last serve of the first series to the initial serve of the second series was less pronounced, *t*(57) = 2.21, *p* = 0.031, *d* = 0.29, 95% CI [0.03, 0.55], and performance levelled off at better values than observed during the first series of serves, resulting in a null effect on aggregate performance. 

**Figure 3:**
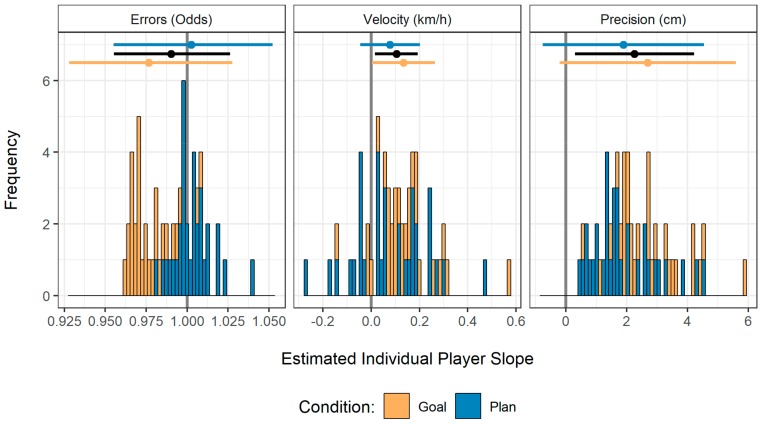
Stacked histograms of the estimated individual player slopes for each performance indicator in the second series of serves, separately for the goal and the plan condition. The upper part displays average slopes along with a 95% confidence interval. The black lines represent an aggregation across conditions.

An evaluation of individual player slopes estimated by the mixed-effects model (Figure 3) reveals that the development of these two indicators was rather homogeneous across players: all of them showed an upward slope in their accuracy and about 80% showed an upward slope in velocity. No development over the second series of serves was observed for the probability of committing errors.

### 3.4. Coach Ratings of Performance Improvements

Coaches rated the performance in the second series of serves as significantly better than the performance in the first series of serves, both in the goal (*M* = 2.5, *SD* = 1.5), *t*(28) = 9.00, *p* < 0.001, *d* = 1.67, 95% CI [1.10, 2.23], and plan condition (*M* = 2.1, *SD* = 1.8), *t*(31) = 6.46, *p* < 0.001, *d* = 1.13, 95% CI [0.68, 1.56]. No significant differences in these coach ratings emerged between conditions, *t*(59) = 1.06, *p* = 0.294, *d* = 0.27, 95% CI [−0.24, 0.77].

## 4. Discussion

We investigated whether and how conveying the self-control strategies of setting goals (goal condition) and making plans (plan condition) to beach volleyball players affects their service performance. To this end, we measured performance (i.e., errors, velocity, and precision) in two subsequent series of serves. After the first series, athletes either set a goal or made a plan to improve their performance based on their coach’s correction instruction. Prior research suggested that both goals and plans should improve performance, although the effects of plans should be more immediate than those of goals due to their automatic nature and thus lead to better aggregate performance (i.e., performance averaged across the serves of the two series of serves). Our data lent only partial support to these hypotheses. We observed the expected initial drops in performance in the goal condition together with a subsequent improvement. However, a similar pattern of results emerged in the planning condition as well. Specifically, we observed an initially decreased aggregate precision in both the goal and the plan conditions during the second series of serves, reflecting a sizeable immediate drop in performance with subsequent recovery. A similar development was observed for velocity, albeit with a smaller drop and a more effective increase afterwards so that aggregate performance was not affected. The concurrent improvement of precision and velocity over time is in line with research showing that people tend to increase their movement velocity as a function of the precision that is required [37]. Note that these decrements in performance cannot be explained by a lack of warm-up. Players engaged in a prolonged warm-up phase under the guidance of their coaches prior to the experiment and no performance changes emerged during the first series of serves. Finally, participants in the plan condition committed fewer errors in the first compared to the second series of serves. However, players in the plan condition also made fewer errors than players in the goal condition during the first series of serves—that is, before we conducted the goal and plan interventions. Accordingly, the observed performance decrement could reflect a regression to the mean, maybe resulting from a randomization failure. What speaks against this interpretation is the observation that the conditions did not differ regarding demographic variables or their experience in playing volleyball. However, we feel that this particular finding should be taken with a grain of salt. Taken together, the observed pattern of results suggests that conveying self-control strategies can hamper service performance.

### 4.1. Implications

This is an important finding for athletes and coaches because both goals and plans represent such fundamental forms of self-control that they are likely to be omnipresent in the sporting context (even though not necessarily as deliberately used strategies). Not only do athletes self-control their actions autonomously during training or competition, correction instructions from coaches and even subtle hints from teammates may trigger self-control in the form of goals or plans. It is thus important to know that correction instructions have no immediate beneficial effect on performance and can even hamper certain aspects of performance. We observed that performance decrements primarily pertained to a decrease in target precision that seemed to reflect initial adjustment costs with subsequent recovery. This indicates that correction instructions may be detrimental during a competition and may require some practice, even for experienced athletes. Contrary to the hypotheses derived from previous research, this practice time could not be eliminated or reduced by plans (versus goals) in the present study.

Our results further suggest that coaches might perceive the effects of goals and plans on their players’ performance as more positive than it seems warranted on the basis of objective performance indicators. There are several explanations for this finding; for instance, their ratings could be based on a positively biased perception of their own correction instructions. Alternatively, the coaches might have focused less on aggregate performance than on the development of performance over time, or maybe myopically on the performance of the single self-controlled motor action that the players focused on. We cannot address this question based on the data we collected. Still, our study suggests that there is a gap between the actual and the perceived effects of self-control interventions that is worth studying.

Although plans in many areas enable more effective and efficient self-control than goals [16] and their effectiveness also appears plausible in the sports context [38], the corresponding research has so far been sparse and yielded contradictory findings [18]. On the one hand, it has been shown that plans can improve competitive performance in tennis [39] and endurance in a physically demanding task [40] compared to goals. On the other hand, there are findings according to which plans in a muscular endurance task do not bring any performance advantages and can even have a negative effect on perceptions of performance [21,35]. In the present study, too, no performance benefits could be identified from plans in comparison to goals. Our study thereby also adds to the literature on costs and benefits of plans in comparison to goals [41] in a naturalistic sport setting with volleyballs serves as a complex behavior that is difficult to regulate.

One possible explanation for the failure to observe a difference between goals and plans comes from research in sport psychology on the acquisition of motor skills. This research has shown that motor skills can be learned better if the attention of the athletes is drawn to the results of the action (external focus) rather than to the performing of the action (internal focus) [42,43]. Consequently, the effectiveness of goals and plans may depend on where they draw athletes’ attention to. Some findings from research on plans generally support this assumption, albeit suggesting that plans can be *more* effective than action goals if they have an internal focus on performing the action [44], which seems to be the case in the field of motor actions as well [45]. The existing literature thus points to potentially complex interactions between the content of goals and plans and their effectiveness in the context of sport. In the present research, we did not provide the content of the goals and plans but rather relied on the coaches’ correction instructions. While this has the advantage of tailoring goals and plans to the individual needs and weaknesses of each athlete in the specific situation, future research should explicitly investigate how goal and plan content—especially in terms of attentional focus—affect their effectiveness in sport.

### 4.2. Limitations

One could argue that the present study would have benefited from a control group without goals and plans. There is no doubt that a comparison with such a control group would have been desirable from an experimental point of view. However, we had limited access to volleyball players and even with the realized design no particularly large test power could be achieved. We thus had to make a judgment call and decided for two of the most commonly investigated self-control strategies [17]. That said, it should also be pointed out that the present sample consists of young athletes with an average of four years of training experience who went through a warm-up phase prior to participating in the study. It is therefore implausible to assume that the changes in performance we observed between the first and the second series of serves merely reflect short-term changes in performance (i.e., unstable performance that might characterize novice players). This argument is especially corroborated by the finding that all three performance indicators were stable across the first series of serves (i.e., no changes occurred regarding errors, velocity, or precision during the corresponding 15 serves). Had the athletes’ performance been unstable, this should have led to changes already during these serves, such as a steady improvement due to further warm-up.

The focus of our present research was on rather immediate, short term-effects of self-control on performance. It would also have been interesting to observe performance over a longer period of time after the intervention. Specifically, our data showed a continuous improvement of precision and velocity throughout the second measurement period. It is not clear how long this trend would have continued and what performance asymptote the athletes might have achieved in further serves; even an eventual improvement cannot be ruled out. Yet, 15 serves can have a decisive impact on the outcome of a competitive game and our results are thus highly relevant for athletes and coaches—also because the first serve in the second series was objectively worst and might thus undermine the coach’s intention to attain immediate improvement at crucial points of a game. A further interesting question pertains to whether goals and plans could have had a stronger impact on performance with a more extensive intervention. We focused on a single action and participants specified a single goal or plan to improve it. In comparison, many other psychological interventions in sport involve comprehensive programs that may last up to several weeks (e.g., self-talk interventions) [46]. This makes it all the more noteworthy how directly goals and plans in the present study affected performance, and it opens up the possibility that conveying self-control strategies for regulating various service-related actions might be a viable alternative to our short intervention.

Another potential route for future research would be to focus on the (perceived) difficulty of performing the motor action that athletes want to control. For instance, it is well-known that the effects of goal and implementation intentions on goal attainment are similar for easy tasks [27], and the failure to observe differences between conditions might results from such an easy task. On the other hand, strong feelings of self-efficacy are crucial for the effectiveness implementation intentions in difficult tasks [47]. Accordingly, one might argue that the present results could in part be explained by insufficient feelings of self-efficacy. Although it seems unlikely that coaches gave correction instructions that were excessively easy or difficult, we cannot rule out that (perceived) task difficulty might explain the present results at least partly.

Finally, future studies might also add more direct measures of the intended or planned behaviors. In our study, for instance, tightening the hand or adjusting stride length in line with the goal or plan might not necessarily have resulted in better objective service performance. This should only be the case if there is a strong relationship between the identified behavior and the objective outcome measures. Our finding that the coaches’ positive assessment of performance development was not reflected in the objective indicators could be taken as an indication for the lack of such a relationship. Furthermore, our focus in the present studies was on framing the coaches’ correction instructions either as goals or plans. While this setup probably best reflects how these instructions are typically conveyed in applied settings, it does not constitute the most powerful intervention study on the effects of goal versus plans [17,18]. Such an intervention study should let all participants set the same goal explicitly in a first step (e.g., to perform a specified behavior) and then instruct participants in the plan condition to generate an if-then plan on top of that.

## 5. Conclusions

In the present research, we investigated whether and how conveying the self-control strategies of setting goals and making plans affect performance in volleyball serves. Our data suggest initial adjustment costs in terms of objective performance (i.e., errors and precision) followed by a recovery period during which performance steadily improved. This is an important finding because goals and plans are likely to be used by athletes and coaches, and could affect performance during competitions when there is insufficient time for performance recovery. However, it is important to keep in mind that these are the results of a pilot study with a small sample and no control group that received neither a goal nor a plan. Further research, for instance using dedicated, high-powered intervention studies, is thus needed to more carefully scrutinize the relationships between setting goals and making plans, the instigated goal-directed behaviors, and objective performance measures in the context of volleyball serves.
